# Professional Justice to the Profession

**Published:** 1844-10

**Authors:** 


*Professional Justice to the Profession.*—There is another kind of discretion and honorable dealing, the want of which has been too often complained of—and it is to be feared, with too much reason; I mean that due regard and kind consideration for the character and feelings of those who may be associated with us in attendance, or who may have preceded us in the treatment of the case.

Under such circumstances I would fain impress upon you this truth, that the best means ever any man adopted to advance his own interest, effectually and permanently, is to be tender of the reputation, and considerate for the interest ot others. There is no surer proof of an honorable mind and conscious integrity of purpose, than a readiness to construe liberally the acts of others; to make allowances for defects, which, after all, may be only apparent, and to palliate errors in others, as far as that can be done consistently with truth, and a due regard to the welfare of those committed to our care. As, then, we value our own charactes, we should be tender of that of others. It is surely wretched architecture to attempt to build a reputation on the crumbling remains of that of another. What can be expected of such a structure, but that it will tumble about the ears of the builder, and perhaps bury him in the ruins. Rest assured that however secretly or cunningly any one may manage, "ambiguas spargere voces," the uncharitable insinuation will be disclosed; for such things, stone walls have ears, or, to use the language of Soloman, "A bird of the air shall carry the voice, and that which hath wings shall tell the matter."

Be assured, sooner or later, those who follow this unworthy course will experience the truth of the Persian proverb, which says that "Curses are like chickens, and always come to roost at home;" and are not insinuations and detractions some of the worst curses of society? Be assured of this, that the ladder by which you can most certainly and safely mount to a high reputation is the good opinion of your professional brethren.

Dr. Cheyne, whose memory is still cherished with reverence by all who knew him, has bequeathed us a memoir of himself well calculated to arrest our attention, not only for the valuable truths which it contains, and the important lessons it conveys, but for the unpretending simplicity with which it is written, and the spirit of pure Christianity with which it is adorned. In this memoir, speaking of the principles of action he adopted, and the means which first led to his advancement, he says: "I endeavored to become acquainted with the characters of those who moved in the highest rank of the profession, and to discover the causes of their success; and I ascertained that, although a man might acquire popularity by various means, he could not reckon upon preserving public favor, unless he possessed the respect of his own profession; that if he would effectually guard his own interests, he must in the first place attend to the interests of others; hence, I was led carefully to study, and liberally to construe that part of medical ethics which regulates the conduct of physicians toward each other;" and, again, having attained to the utmost limit of success, he observes: "By a good arrangement, punctuality, attention to the interests and feelings of my professional brethren, and by prudence, the means which had apparently led to my advancement, I now tried to avoid those reverses to which my professional life is ever subject." And many here know the result, that when he left us in the autumn of his days, when "his way of life had fallen into the sear, the yellow leaf," he took with him "golden opinions from all sorts of people"—and possessed, in rich abundance, what the guilty monarch wept to anticipate the want of—

"As honor, love, obedience, troops of friends."

And here, I feel persuaded, that it has already occurred to many of you, that it were only necessary to change the name, and all these sentiments of admiration and regard would equally apply to another, who just now, "after life's fitful fever, sleeps well," and if I speak of one who, after a long life of honor and utility, carries with him to the grave the affectionate respect and reverence of all who knew him—one so entwined with our best and tenderest feelings and recollections, that the poet's exclamation will not appear extravagant:—

"Ah quanto minus,

Versari cum aliis, quam meminisse tui."

When I say, I speak of such a one, I feel that your hearts anticipate the application of my words, and murmur the name of Colles.—*Address before the Dublin Obstetrical Society, by Dr. Montgomery.*—(*Ibid.*)

